# Preauricular Sinus: A Tale of Forgetful Rediscovery

**DOI:** 10.7759/cureus.8885

**Published:** 2020-06-28

**Authors:** Boyko Matev, Emran Lyutfi, George S Stoyanov, Nikolay R Sapundzhiev

**Affiliations:** 1 Medicine, Medical University of Varna, Varna, BGR; 2 General and Clinical Pathology/Forensic Medicine and Deontology, Medical University of Varna, Varna, BGR; 3 Otolaryngology, Medical University of Varna, Varna, BGR

**Keywords:** preauricular sinus, medical history, genetic syndromes, medicine in art

## Abstract

The preauricular sinus (PAuS) is a congenital foramen, opening or invagination, usually located on the crus of the auricular helix and is considered a congenital malformation and component of multiple syndromes. The structure can be present unilaterally or bilaterally, with the possibility of more than one fistula present on one ear, predominantly on the auricular tags. As a well-defined and established clinical entry, PAuS has a very strictly laid-out history. However, different works of art give us a glimpse into the structure before its first true clinical description, showing that the PAuS was known to man long before it was first clinically described, such as those of Hieronymous Bosch, with the first medical descriptions being attributed to Heusinger and Virchow. In modern times, the condition is considered both an individual malformation and a component of several genetic syndromes.

## Introduction and background

The preauricular sinus (PAuS) is a congenital malformation characterized by a dent, dimple, or a foramen, usually located on the crus of the auricular helix [1-2]. Rarely structures with the same characteristics and clinical course can be found on the pinna, tragus, or even in the postauricular area contradictory to the established name in modern times [1-3].

The structure can be present unilaterally or bilaterally, with the possibility of more than one fistula present on one ear, predominantly on the auricular tags [3-6]. The structure presents as a simple epithelial invagination into the auricular stroma and can often become inflamed, leading to discharge from the superficial opening and discomfort [1,7]. This itself can lead to serious complications, such as facial paralysis, due to its location near the facial nerve [1,7,8]. Clinical history in such cases is important to distinguish between a fistulized abscess and an inflamed PAuS. Furthermore, chronic inflammation is a risk factor for cancerogenesis, and thus a squamous cell carcinoma ex PAuS (carcinoma from a PAuS) may develop later in life [9-11]. This is the reason why the structure evokes the interest of many otolaryngologists, specialists, and pediatricians.

As a well-defined and established clinical entry, PAuS has multiple contributors in the medical field that helped define it and its significance. However, different works of art give us a glimpse into the structure before its first true clinical description, showing that the PAuS was known to man long before it was first clinically described.

## Review

Artistic representation

**Figure 1:**
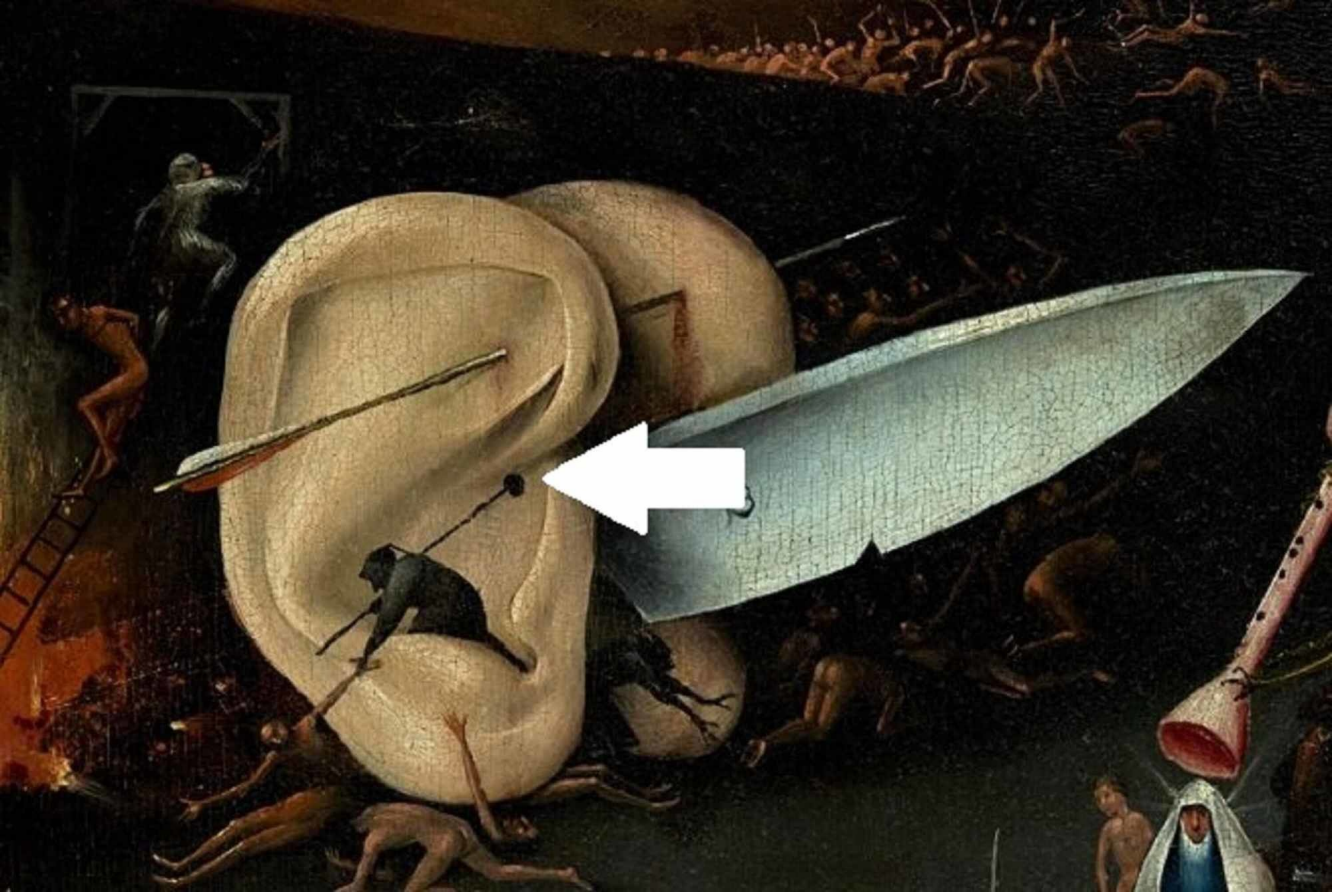
Hieronymous Bosch, Garden of Earthly Delights, a magnified section of the right panel of the interior section (Hell). The grotesque ear has a structure reminiscing of a PauS (arrow). PAuS, preauricular sinus

Hieronymus Bosh (c. 1450-1516) was a Dutch artist whose most famous painting is “The Garden of Earthly Delights”. The painting illustrates exterior and interior panels [11]. The exterior panel resembles the creation of the world, whereas the interior consists of three panels that represent, respectively, The Garden of Eden (left panel), The Human World (middle panel), and The Judgment Day or Hell (right panel). A small segment of the right panel illustrates a pair of ears on which a structure resembling a PAuS is seen (Figure 1) [12].

First medical descriptions

The PAuS was first described in 1864 by C. F. Heusinger when describing the findings in a patient characteristic of the brachio-oto-renal (BOR) syndrome [13]. He detailed his finding and also referred to several already described phenomena that seemed detached from each other, such as the preliminary work by Dzondi, who described and defined congenital tracheal fistulas [13]. It is important to note that the auricula entity described by Heusinger, although morphologically identical, is its separate entity from the cervical and other brachial fistulas described before his observations [13,14].

**Figure 2:**
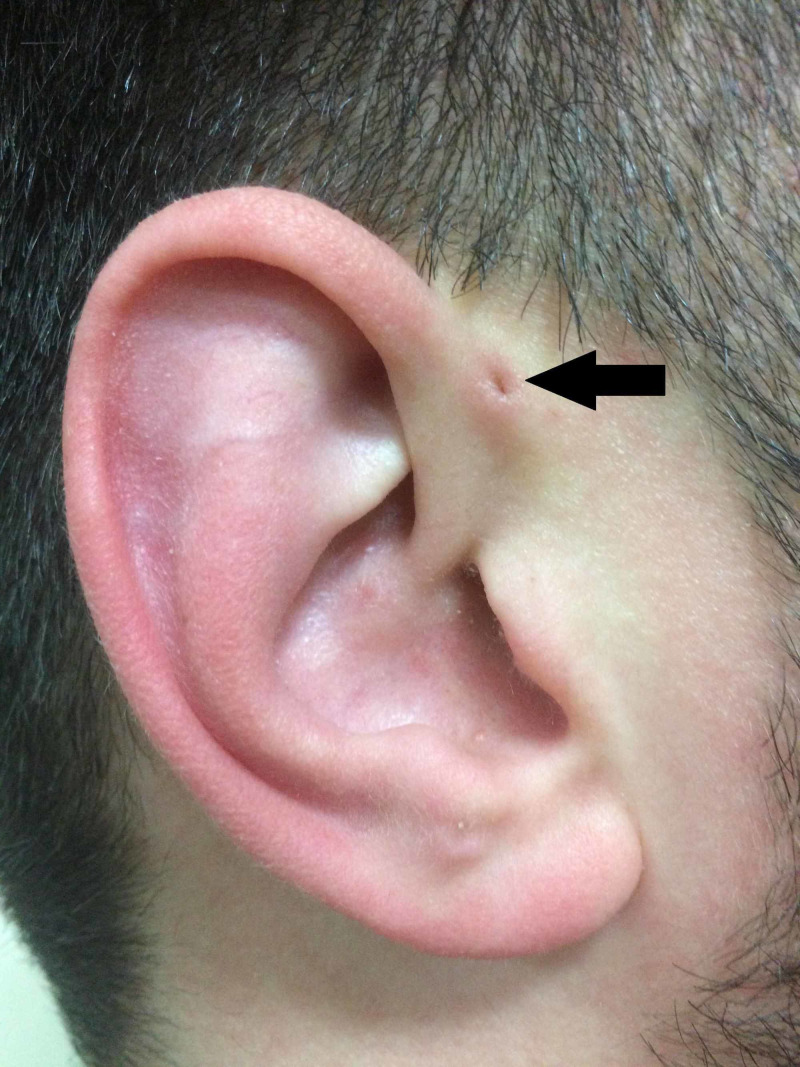
Classical representation of PAuS (arrow). PAuS, preauricular sinus

Following Heusinger's description, Rudolph Virchow (1821-1902) also described the structure in 1864, although simply stating in his article “I also know a patient like that” [14]. Virchow, however, was the first to postulate that the PAuS is a result of a defect in the embryological fusion of the pharyngeal arches, a statement that was widely contradicted at the time (Figure 2).

**Figure 3:**
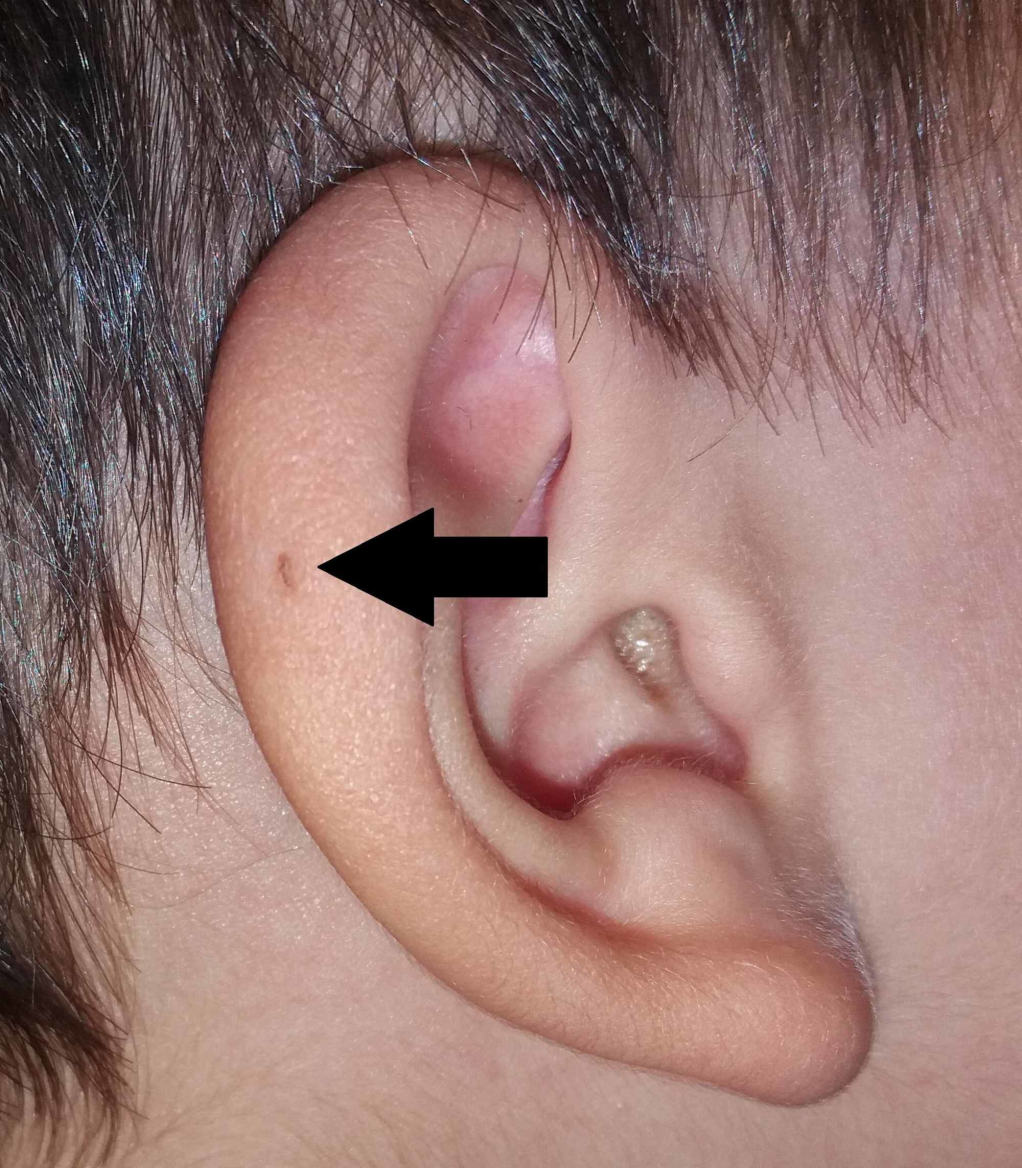
Variant of PAuS on the auricular helix (arrow). PAuS, preauricular sinus

Sir James Paget also published on the subject in 1878, describing patients with such fistulae present not only on their ears but also on their neck [15]. Based on the description of his patients, he coined the term oto-branchial fistula [15] (Figure 3). Sir James Paget widely adapted the viewpoints of Virchow’s theory of the origin of PAuS.

During the first part of the 20th century, the PAuS became known as “natural earring holes” and “fistula auris congenita”, with several authors publishing on the subject, including its pattern of inheritance [3,15,16]. The phenomena and its transgenerational inheritance allowed for an extensive set of studies and theories on its cause, widely converging on Virchow’s original statements [17-23].

One of the most important contributions from that time period was in 1955 by Fourman and Fourman, who researched the inheritance of PAuS, constructing a genealogical tree, focusing on the inheritance of the structure [24]. They concluded that half of the siblings of the affected family have the structure and thus postulated that the sinus is inherited as a dominant trait, but with “incomplete penetrance” due to it skipping in generations or individuals [24].

Despite that initial interest, however, little to no articles were published after the late 1940s, until a small resurgence in later years, where the structure reappears under a new name - PAuS. Since then, research on the structure has seldom been carried out, and the mechanisms of its transgenerational inheritance remain widely forgotten in the literature of the 1930s and 1940s.

PAuS-associated syndromes

Melnick-Fraser Syndrome

The syndrome was first described in 1864 by Heusinger [13]. However, in 1975, Melnick et al. described a series of patients with it, and in 1980 Fraser et al. described another series of cases with the hallmarks of the syndrome in a specialized school for the deaf [25,26]. The condition is considered relatively rare, with 250 new cases of Melnick-Fraser being diagnosed in Japan in 2014 [27]. First believed to be a variant of BOR syndrome, later on, Melnick widened the syndrome into a group of nearly identical conditions dependent on the presence or absence of different hallmarks [28,29]. The term Melnick-Fraser syndrome was later coined to unify the characteristics of the separate syndromes and to completely separate it from Fraser syndrome, isolated urogenital malformations, and Frasier-Lynch syndrome, familial colorectal polyposis, with which BOR had quite often been confused as a term at that point in time. Thus, sadly Heusinger’s contribution has been widely forgotten.

Beckwith-Wiedemann Syndrome​​​​​​​

The condition was originally described in 1963 by American pathologist John Beckwith as a combination of exomphalos, macroglossia, and gigantism, therefore referred to as exomphalos-macroglossia-gigantism (EMG) syndrome (Poster: Beckwith, JB: Extreme Cytomegaly of the adrenal Fetal Cortex, Hyperplasia of the Kidneys and Pancreas, and Leydig-Cell Hyperplasia : Another Syndrome?. Annual Meeting of Western Society for Pediatric Research, Los Angeles, CA, November 11, 1963). In 1964, however, independently of John Beckwith, the German pediatrician Hans-Rudolph Wiedemann also described several patients with the same hallmarks of the disease but also included symptoms such as adrenal hyperplasia [30]. Over time, the syndrome was dubbed Beckwith-Wiedemann syndrome, and the diagnostic criteria were expanded to include PAuS, hyperplastic kidneys, microcephaly, neonatal hypoglycemia, and hepatoblastoma developing later in life [31,32]. Mutations in 11p15 involving genes such as insulin-like growth factor 2 (IGF-2), cyclin-dependent kinase inhibitor 1C (CDKN1C), H19, and potassium voltage-gated channel subfamily Q member 1 overlapping transcript 1 (KCNQ1OT1) have been established in such patients [32]. The mechanism of inheritance is still undefined as in several patients with this condition, the exact transgenerational inheritance could not be determined, deeming it as a sporadic recessive defect. Beckwith-Wiedemann syndrome and its hallmarks are especially important to be distinguished in patients conceived with in vitro fertilization as the incidence in that population is much higher [33-35]. Beckwith-Wiedemann is considered a rare condition with an incidence of 1 per 13,700 children born, accounting for a total of about 300 children born with Beckwith-Wiedemann in the United States of America annually [36].

Lachiewicz-Sibley Syndrome​​​​​​​

First described in 1985 by Lachiewicz et al., this is one of the rarest syndromes ever described [37]. The syndrome is very similar to Melnick-Fraser, though only PAuS and hypoplastic kidneys with early-onset proteinuria were found in the descendants of British and Irish immigrants settling in Ohio in the 1800s and later Nebraska. At the time of the original study, from 130 living relatives, 12 members had PAuS and hypoplastic kidneys, 10 had only hypoplastic kidneys, and 3 had only PAuS [37]. Although the exact locus of mutation remains unknown, the condition is inherited in an autosomal dominant fashion.

Twenty-first century

Today although still relatively under-researched, the PAuS is a common clinical entity with well-defined clinical strategies for treatment. Some studies have identified a genetic association between PAuS and the 8q11.1-13.1 locus; however, the results have not been reproduced widely [1,5,7,38]. Other than this individual study, no other studies have focused on the genetic reason for PauS nor have associated it with the aforementioned genetic syndromes, with genealogic studies are few and far in-between [16,24,39].

The clinical course has been well described, and treatment strategies including predominantly surgical excision have been well established in modern otorhinolaryngology and head and neck surgery [5,7,8,40]. Nowadays, patients with PAuS, not associated with a genetic syndrome, undergo a safe intervention process with few described complications, whereas patients with genetic syndromes undergo the same curative modalities, and the excision of the PauS does not affect their overall clinical course [7,40-42].

## Conclusions

The PAuS, as it was first described, is often a component of several eponymous inherited syndromes such as Melnik-Fraser (BOR syndrome), Beckwith-Wiedemann, and Lachiewicz-Sibley, which also include a variety of constant kidney and other distant variable malformities such as cysts, fistulae, facial and neural defects, and others. These syndromes relatively rarely underline the importance of Virchow’s original statement that the condition is caused by malfusion of pharyngeal arches, a process taking place parallel to some stages of kidney development. However, there is no evidence in the scientific literature inferring that the presence of PAuS always concludes to the presence of any of the aforementioned syndromes. Although the exact model of inheritance of the PauS, according to modern understandings and its genetic and molecular hallmarks, are yet to be fully determined and described, some authors still consider it as an irregular dominant mutation with reduced penetrance.
